# Safety of endoscopic procedures with monopolar versus bipolar instruments in an ex vivo porcine model

**DOI:** 10.1186/s12876-020-1176-9

**Published:** 2020-01-31

**Authors:** Kensuke Shinmura, Hiroaki Ikematsu, Motohiro Kojima, Hiroshi Nakamura, Shozo Osera, Yusuke Yoda, Keisuke Hori, Yasuhiro Oono, Atsushi Ochiai, Tomonori Yano

**Affiliations:** 1 Department of Gastroenterology and Endoscopy, National Cancer Center Hospital East, 6-5-1, Kashiwanoha, Kashiwa, Chiba, 277-8577 Japan; 2 Department of Pathology and Clinical Laboratories, National Cancer Center Hospital East, 6-5-1, Kashiwanoha, Kashiwa, Chiba, 277-8577 Japan

**Keywords:** Colorectal endoscopic resection, Monopolar instrument, Bipolar instrument

## Abstract

**Background:**

Monopolar instruments are generally used in colorectal endoscopic mucosal resection (EMR). Bipolar instruments have previously been reported to be as safe as monopolar instruments. We sought to compare the safety of the monopolar and bipolar snare and hemostatic forceps in an animal model.

**Methods:**

We created 5-mm, 10-mm, and 15-mm target lesions on an ex vivo porcine rectum. Two lesions of each size were resected via monopolar polypectomy (M-P), monopolar EMR (M-E), bipolar polypectomy (B-P), and bipolar EMR (B-E). We performed a pathological evaluation of the conditions of perforation and the effects of burning on the tissues. In addition, we burned the muscularis propria covered with submucosal layer using monopolar and bipolar hemostatic forceps and performed pathological evaluations.

**Results:**

Polypectomy and EMR were performed in a total of 24 target lesions. A perforation was found on histology in one case of M-P and one case of M-E after removing target lesions of 15 mm in diameter. There were no perforations during endoscopic resection using the bipolar snare. The thermal denaturation in B-P did not reach the muscularis propria layer regardless of the size of the target lesion. Although thermal damage after using monopolar hemostatic forceps was extensive, thermal denaturation was only seen on the surface of the submucosal layer when bipolar hemostatic forceps were used.

**Conclusions:**

Bipolar instruments cause less damage to the tissue than monopolar instruments. Our results also suggest that bipolar instruments may be safer than monopolar instruments in endoscopic procedures for colorectal lesions.

## Background

Colorectal cancer (CRC) is a major cause of morbidity and mortality worldwide. It is important to perform colonoscopy screening to decrease CRC-related mortality. Reports have shown that detection and resection of adenomatous polyps via screening and therapeutic endoscopy prevent the CRC-related adenoma-carcinoma sequence [1, 2].

Endoscopic resections (ER) including cold or hot biopsy, polypectomy, endoscopic mucosal resection (EMR), and endoscopic submucosal dissection (ESD) for colorectal lesions have been recently and widely performed. It is possible to perform polypectomy and EMR without hospitalization. However, patients occasionally experience complications such as bleeding and perforation after ER, even when small lesions are resected [3–8]. Cold snare polypectomy for colorectal lesions smaller than 10 mm has recently been reported to be safe and effective, but it is controversial for lesions larger than 10 mm [9]. ER for colon lesions larger than 10 mm can be performed using either a monopolar or bipolar snare; the monopolar snare has generally been used in resections. In addition, although there are many reports on the effectiveness of monopolar hemostatic forceps for upper gastrointestinal bleeding [10–13], very few reports exist on hemostatic forceps for lower gastrointestinal bleeding, except for during ESD.

With the monopolar device, the current passes from the active electrode to the target lesions through the patient’s body and finally exits the patient via a return electrode. With the bipolar device, the current only passes through the tissue between the two electrodes of the instrument [14, 15]. More convenient and safer modes of treatment have been sought for resection of colorectal lesions larger than 10 mm and for bleeding because the digestive wall is thin, especially in the wall of the colon. However, few reports have compared the safety of the monopolar and bipolar snare and hemostatic forceps.

In this study, we compared the safety of monopolar and bipolar instruments for colorectal polypectomy, EMR, and hemostasis in an animal models.

## Methods

### Polypectomy and EMR

To perform graded diameter resections, we created 5 mm, 10 mm, and 15 mm target lesions on an ex vivo porcine rectum (Tokyo Shibaura Zouki Co., Ltd., Tokyo, Japan). Two lesions of each size were resected using monopolar polypectomy (M-P), monopolar EMR (M-E), bipolar polypectomy (B-P), and bipolar EMR (B-E). An electrosurgery generator unit (ICC200; ERBE Elektromedizin GmbH Co., Ltd., Tubingen, Germany) was used for all resections. We used the monopolar snare (Captivators; Boston Scientific, Boston, MA, USA) with the cutting mode and the bipolar snare (Dragonare; Xemex Co., Ltd., Tokyo, Japan) with the forced coagulation mode at the same settings as those used in humans. The cutting mode was set at 120 W in the endo cut mode, and the coagulation mode was set at 15 W. A return electrode was directly pasted on the outside of the porcine rectum during EMR. Before EMR, sufficient volume of saline was injected into the submucosal layer located under the target lesion. The volume of saline injected were not measured.

**Fig. 1:**
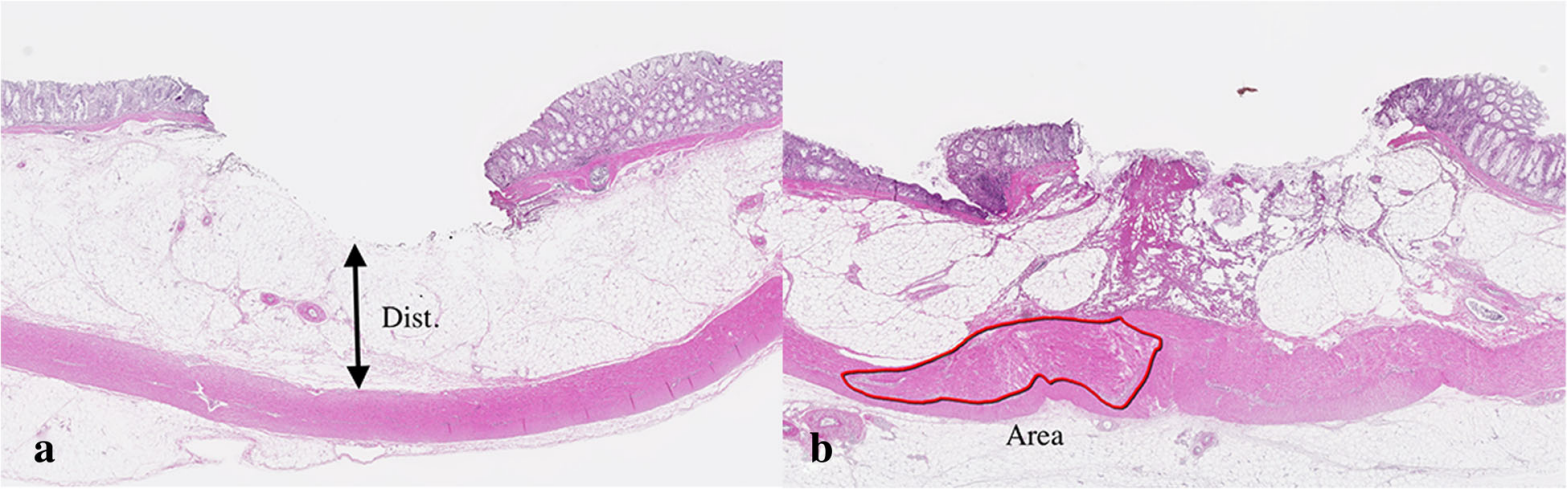
Histopathology (H-E stain) of the target lesion. (**a**) Distance is from the muscularis propria to the thermal denaturation of the submucosal layer. (**b**) Area shows tissue damage to the muscularis propria. Dist, distance

We evaluated the tissue remaining after endoscopic resection for the pathological perforation and the effects of burning. We made the following measurements of the effects of burning (Fig. 1): 1) distance from the muscularis propria to the thermal denaturation of the submucosal layer (Dist); and 2) area of tissue damage to the muscularis propria (Area). The term “thermal denaturation” was defined as the pathological change of normal structure such as fusion of connective tissue in submucosal layer. The term “tissue damage” was defined as pathological change of normal structure such as destruction of sequences in muscularis propria. The effects of burning means the thermal denaturation and/or tissue damage.

### Hemostasis

We used the same porcine rectum specimen that was used for polypectomy and EMR. The mucosa of the porcine rectum was resected with scissors, and the submucosal layer was exposed.

Three target points on the exposed submucosal layer, which were continuously cauterized for 5, 10, or 15 s using the monopolar hemostatic forceps (Coagrasper: FD-410LR; Olympus, Tokyo, Japan); a second set of three target points were cauterized by the same method using the bipolar hemostatic forceps (Tighturn: Xemex Co., Tokyo, Japan). The target points were then pathologically evaluated. An electrosurgery generator unit was set at 60 W in soft coagulation mode for the monopolar device and at 35 W in the soft bipolar mode for the bipolar device.

All the endoscopic procedures were performed by two experienced endoscopists and the specimens were histologically evaluated by the same pathologist.

## Results

### Polypectomy and EMR

**Fig. 2:**
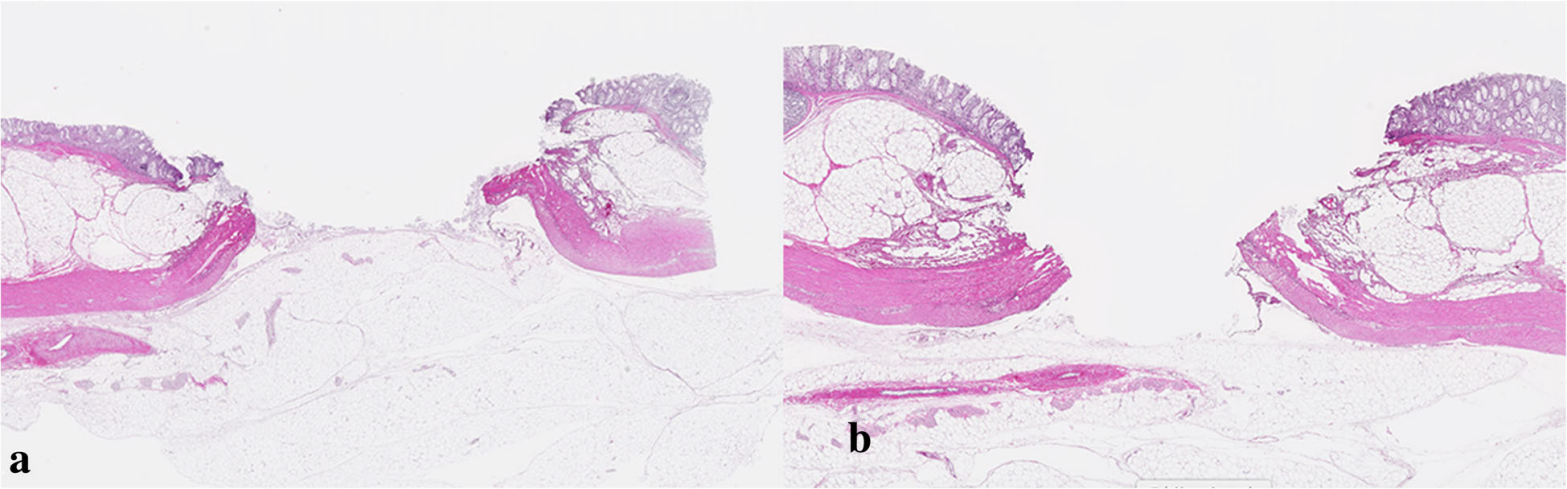
Histopathology (H-E stain) of the target lesion with a diameter of 15 mm removed with a monopolar snare. (**a**) Histopathology after polypectomy using a monopolar snare. (**b**) Histopathology after EMR using a monopolar snare. Both A and B show cases with perforation

**Fig. 3:**
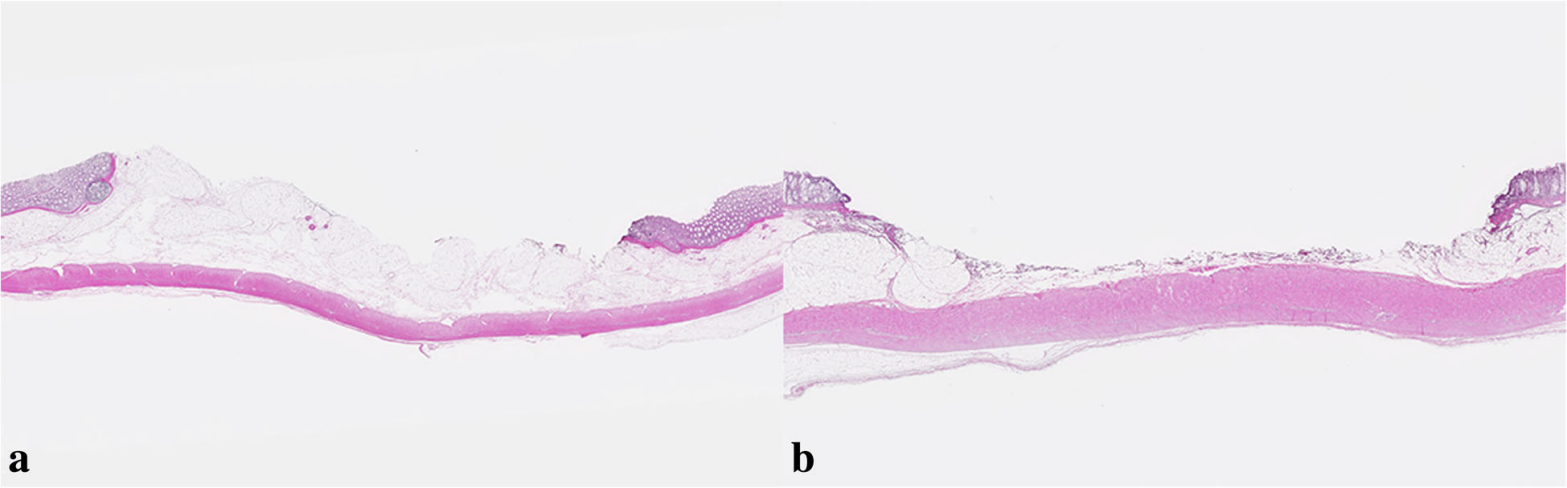
Histopathology (H-E stain) of the 15-mm target lesion removed with a bipolar snare. (**a**) Histopathology after polypectomy using a bipolar snare. (**b**) Histopathology after EMR using a bipolar snare

The results are shown in Table 1. EMR and polypectomy were performed in 24 target lesions. A perforation was found on pathological analysis in one case of M-P and one case of M-E after removing target lesions 15 mm in diameter (Fig. 2). Conversely, there was no perforation in cases undergoing endoscopic resection using the bipolar snare. While the thermal denaturation in B-P did not reach the muscularis propria layer regardless of the size of the target lesion (Fig. 3), tissue damage without perforation was observed in the muscularis propria layer in most cases of M-P (4 in 5 cases, 80%). The tissue damage to the muscularis propria was detected in lesions larger than 10 mm in diameter in M-E and B-E.

**Table 1:** Tissue damage due to endoscopic resection using a monopolar or bipolar snare

Size of the target lesion	Monopolar		Monopolar		Bipolar		Bipolar	
	polypectomy		EMR		Polypectomy		EMR	
	Dist	Area	Dist	Area	Dist	Area	Dist	Area
	(mm)	(mm^2^)	(mm)	(mm^2^)	(mm)	(mm^2^)	(mm)	(mm^2^)
5 mm	–	0.09	0.1	0	1.18	0	0.36	0
5 mm	0.6	0	0.2	0	1.05	0	0.18	0
10 mm	–	1.06	–	0.88	0.4	0	0.08	0
10 mm	–	1.36	–	0.77	1.3	0	–	0.24
15 mm	–	P	–	P	1.35	0	–	0.06
15 mm	–	2.56	–	0.66	0.93	0	–	0.07

*Dist*, distance from the muscularis propria to the thermal denaturation of the submucosal layer; *Area*, area of tissue damage to the muscularis propria; *P*, perforation; −, unmesurable

### Hemostasis

**Fig. 4:**
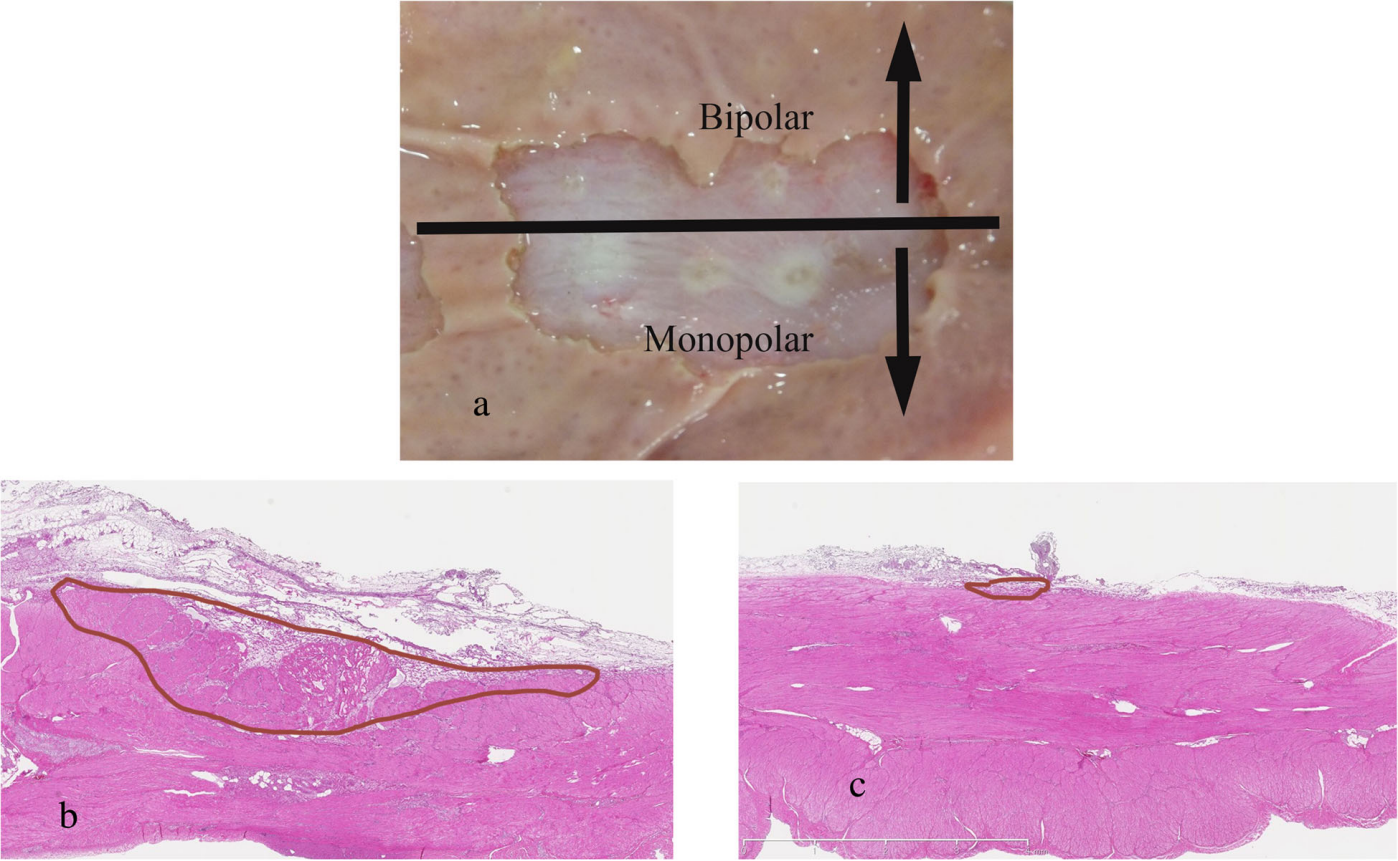
Exposed muscularis propria burned by monopolar hemostatic forceps or bipolar hemostatic forceps. (**a**) The three burning points of the upper row were made using bipolar forceps, and the thee burning points of the lower row were made using monopolar forceps. (**b**) Histopathology of the burning point using monopolar hemostatic forceps (15 s). (**c**) Histopathology of the burning point using bipolar hemostatic forceps (15 s)

The results are shown in Fig. 4. Tissue damage caused by monopolar hemostatic forceps reached the muscularis propria at all timepoints (5, 10, and 15 s). In contrast to monopolar hemostatic forceps, thermal denaturation was found only in the part of the submucosal layer that was grasped using the bipolar device. Tissue damage was rarely detected elsewhere at all timepoints (5, 10, and 15 s).

## Discussion

In the present study, endoscopic resection and hemostasis using a bipolar instrument was safer than that performed using a monopolar instrument in an ex vivo animal model. To our knowledge, this is the first study comparing the safety of monopolar instruments with bipolar instruments from the viewpoint of pathological evaluation in animal colon models.

Endoscopic resection of colorectal polyps is a widely used form of minimally invasive surgery. Almost all previous reports of complications have been described regarding ER with a monopolar snare. Panteris et al. reported that the rate of perforation during therapeutic endoscopy in the colorectum, including ESD, was 0.2–5% [16]. The rate of perforation during polypectomy and EMR using a monopolar snare was 0–0.1% [17, 18] and 0.4–1.5% [19, 20], respectively.

One way to reduce complications during therapeutic endoscopy is the use of a bipolar instrument. This device theoretically minimizes the depth of burning and degree of tissue destruction [21, 22]. Saraya et al. reported an incidence of perforation as low as 0.08% for endoscopic resection with a bipolar snare. They also found that a bipolar snare appeared to be at least as safe as a monopolar snare for endoscopic resection of colorectal polyps with regard to the associated perforation rate [4]. In the current study, polypectomy and EMR with a monopolar snare caused one perforation, respectively. However, polypectomy with a bipolar snare did not damage the muscularis propria and resulted in no perforations. Tissue damage to the muscularis propria was detected in target lesions larger than 10 mm in diameter in polypectomy and EMR with a monopolar snare and EMR with a bipolar snare. Therefore, we surmise that lesions smaller than 10 mm in diameter can be safely resected and lesions of 10–15 mm can be resected more safely with polypectomy with a bipolar snare than with a monopolar snare. If lesions in 10–15 mm in diameter need to be resected by EMR, a bipolar snare would be first choice.

When comparing bipolar EMR with bipolar polypectomy for 10-15 mm target lesion in this study, bipolar EMR causes more tissue damage than polypectomy in terms of distance of intact submucosa and area of muscularis propria damage. In our opinion, there is a possibility that as a result of pressing the soft bipolar snare on the saline-injected mucosa, excessive force is applied in the vertical direction, leads to the compression of the muscularis propria during bipolar EMR. Therefore, thermal energy may reach the muscularis propria during bipolar EMR. In contrast, the bipolar snare can gently pull the mucosa vertically during bipolar polypectomy. Therefore, thermal energy may not reach the muscularis propria. It is also possible that thermal efficiency is related to the amount of saline injected.

In this study, the cutting mode is different between the monopolar (endo cut mode) and bipolar snares (coagulation mode). Chino et al. reported that the blend mode (PSD-20 model; Olympus, Tokyo) was better than the pure cut or coagulation modes when using a monopolar snare in polypectomies [23]. We safely performed polypectomies and EMR with the coagulation mode using the bipolar snare in the porcine model. If the bipolar snare is used with the same setting as that used in the monopolar snare, it can lead to half-burned tissue and may cause bleeding. When using the bipolar snare, we used the mode in which coagulation was most likely to occur, avoiding the pure cut mode and lowering the output setting when using the monopolar snare.

Endoscopic hemostasis for lower gastrointestinal bleeding is mainly performed by clipping or coagulation [24–26]. With gradually increasing EMR or ESD, safe and effective endoscopic hemostasis is required for therapeutic endoscopy. Although most endoscopists do not generally use clipping for active bleeding during endoscopic procedures, especially ESD, they tend to use monopolar instruments for active bleeding. Kataoka et al. evaluated monopolar and bipolar hemostatic forceps using swine gastric mucosa specimens and revealed more intense thermal denaturation of the surrounding and deep tissue with monopolar hemostatic forceps than with bipolar hemostatic forceps [27]. Monopolar forceps combined with pure coagulation current have been shown to produce deeper tissue injury and higher rates of histologic transmural damage in porcine and canine animal models [23, 28]. In our study, we demonstrated that there was minimal tissue damage to the muscularis propria using bipolar hemostatic forceps. We believe that bipolar hemostatic forceps are suitable for treating active bleeding or penetrating artery vessels in the muscularis propria.

The bipolar instrument is advantageous because it can be used for endoscopic resection without application of a return electrode and can eliminate the possibility of return electrode burn and contralateral colorectal wall burn, thus reducing cost and time. Furthermore, endoscopic procedure with the bipolar instrument can be conducted safely for patients with implanted metal objects such as pacemakers, artificial joints, and accessories.

In recent years, many reports have showed the safety of cold snare polypectomy (CSP) for small colorectal polyps (< 9 mm) [9, 29]. Although CSP has few complications, compared with hot monopolar snare polypectomy for small polyps, it is unclear whether resecting ≥10 mm polyps using CSP is safe or not. We believe that resecting 10–15 mm polyps using hot bipolar snare polypectomy or EMR could be a treatment option based on the safety data produced in this study.

We acknowledge that this study has several limitations. First, We could not use live animals, as there was a possibility that accurate pathological evaluation of the effects of burning could not be performed. This was because the mucosal defect would change to a larger ulcer at least 24 h after polypectomy [30]. We could not investigate these ulcers, as it would be difficult to take the in vivo reaction to electrocautery into account. Second, The perforation rates of endoscopic procedures using monopolar snare in the current study was higher than the previous reports [17–20]. In clinical EMR, we take the following process; 1) saline-injection, 2) deflating air in colon to grasp mucosa easily, 3) snaring, 4) slightly releasing the snare and inflating air to avoid grasping muscularis propria, and 5) snaring firmly and cutting. In the current study, it was impossible to use the technique of deflating or inflating air because of an ex vivo porcine model. Therefore, there was a possibility to grasp muscularis propria when snaring and cutting. Further, a hemostatic forceps was used on the muscularis propria layer, which did not show active bleeding. However, the point we wanted to clarify was not the hemostasis, but the influence of hemostasis on tissues. In addition, the current study had a small sample size and we could not statistically evaluate whether monopolar instruments or bipolar instruments were safer. However, bipolar instruments tended to be safer. A larger analysis of human data samples for comparing monopolar and bipolar instruments is needed in the future.

## Conclusions

We demonstrated that bipolar snares cause less damage to the tissue than monopolar snares, especially the muscularis propria, for target lesions 10–15 mm in diameter and bipolar hemostatic forceps less damage to the tissue than monopolar forceps, although an ex vivo porcine rectum was used. We believe that bipolar instruments are safer than monopolar instruments for colorectal polypectomy, EMR, and hemostasis.

## Data Availability

The datasets used and analyzed during the current study will be available from the corresponding author on reasonable request.

## Abbreviations

B-E: Bipolar endoscopic mucosal resection
B-P: Bipolar polypectomy
CRC: Colorectal cancer
CSP: Cold snare polypectomy
EMR: Endoscopic mucosal resection
ER: Endoscopic resection
ESD: Endoscopic submucosal resection
IRB: Institution review board
M-E: Monopolar EMR
M-P: Monopolar polypectomy
